# Feasibility trial protocol assessing the use of aerobic exercise to promote recovery from work-related concussion

**DOI:** 10.1371/journal.pone.0325701

**Published:** 2025-06-12

**Authors:** Jacob I. McPherson, Jeffrey C. Miecznikowski, Haley Chizuk, Patrick Sparks, John J. Leddy, Mohammad N. Haider, Christopher J. Stavisky

**Affiliations:** 1 Department of Rehabilitation Science, School of Public Health and Health Professions, State University of New York at Buffalo, Buffalo, New York, United States of America; 2 Department of Biostatistics, School of Public Health and Health Professions, State University of New York at Buffalo, Buffalo, New York, United States of America; 3 Department of Orthopedics, Jacobs School of Medicine and Biomedical Sciences, State University of New York at Buffalo, Buffalo, New York, United States of America; PLOS: Public Library of Science, UNITED KINGDOM OF GREAT BRITAIN AND NORTHERN IRELAND

## Abstract

**Introduction:**

Work-related concussion (WRC) is a common occupational injury that has received far less research attention compared to sport-related concussion (SRC). WRC results in prolonged lost time claims, adversely influences perceived life roles, and negatively impacts personal and family finances. WRC also affects workforce capacity in industries that are already facing worker shortages (e.g., construction, manufacturing, healthcare, etc.). Targeted heart rate aerobic exercise (THRAE) has emerged as an effective treatment for speeding recovery from SRC and for reducing the incidence of persisting post-concussive symptoms (PPCS). The aim of this proposed study is to explore the feasibility of THRAE in adults with WRC as there are very few interventions that have been rigorously studied in this population.

**Materials and Methods:**

We describe a feasibility trial to implement THRAE in individuals with WRC. While this study will not test specific hypotheses related to effectiveness, it will provide key information related to adherence, safety, and psychosocial factors related to adherence and participation. Additionally, this study will provide mean and standard deviation values for the measures applied to this novel population to inform power analyses for larger-scale trials.

**Trial registration:**

Clinicaltrials.gov NCT06263179

## Introduction

Concussion, a term often used synonymously with mild traumatic brain injury (mTBI), results in complex pathophysiologic processes causing a disturbance in brain function [1]. The estimated worldwide yearly incidence of mTBI is nearly 42 million cases and accounts for 70–90% of traumatic brain injuries [2]. Recent data from the Centers for Disease Control reveal that approximately 22,000 emergency department visits for WRC occurred in 2020 [3]. The WRC incidence rate of 31.8/100,000 is notably larger than some reports of SRC incidence [4, 5, 6]. The Occupational Safety and Health Administration states that the direct cost of a single WRC is $54,571, which translates to an annual expenditure of at least $1.2 billion [7].

There has been substantial growth in the literature describing pediatric, adolescent, and adult athletes who suffer SRC. This has led to a deeper understanding of mTBI and has prompted the development of clinical management guidelines specifically for SRC [8, 9, 10]. Unfortunately, evidence-based treatments for individuals with WRC are far more limited. In addition to a highly productive research program, the Concussion Management Clinic and Research Center (CMCRC) at The University at Buffalo (UB) is a key provider of clinical services to individuals with WRC in the Buffalo region. Data from the CMCRC indicate that adults with WRC take more than 8 times longer to recover from a concussion when compared with individuals with SRC [11]. Prior studies suggest that the longer injured workers are out of work, the less likely they are to rejoin the workforce [12]. Not only does this have tremendous financial implications for workers and their families, but WRC disproportionally affects several key industries that are already experiencing worker shortages including construction, manufacturing, healthcare, and service [13]. Accelerating return to work (RTW) for individuals with WRC has immediate individual psychological and economic benefits, supports a stable workforce, and may also reduce business expenses related to workers’ compensation insurance costs.

Targeted heart rate aerobic exercise (THRAE) is an effective treatment to speed recovery from SRC and to reduce the incidence of persisting post-concussive symptoms (PPCS) among athletes and adolescents [14,15]. Similar active interventions have not been rigorously studied in the WRC population. Here, we describe a feasibility trial designed to gather data on the safety and adherence of a THRAE program, and to help understand potential psychosocial factors influencing exercise behavior (e.g., workplace satisfaction, anxiety, depression).

## Materials and methods

We intend to complete a prospective feasibility trial conducted at an outpatient sports medicine clinic led by a board-certified sports medicine physician. Ethics approval for this project has been obtained from the University at Buffalo Institutional Review Board (FWA00008824). This protocol has been registered at the ClinicalTrials.gov Protocol Registration and Results System (ID: NCT06263179) and can be found here: https://clinicaltrials.gov/study/NCT06263179.

### Study design and participants

To be eligible to participate in this study, a patient must have a physician-confirmed diagnosis of a concussion within 6 weeks of the injury. Diagnosis is based on history symptoms since injury, identification of impairments on physical examination (Buffalo Concussion Physical Exam [BCPE [16]]), and responses on a validated symptom profile (Post-Concussion Symptom Scale [PCSS [17]]). Patients diagnosed with WRC who meet eligibility criteria will have the study explained to them and will be asked to provide written consent to participate by the study’s research assistant (RA). Participants will then be provided with a Physical Activity Readiness Questionnaire for Everyone (PAR-Q+), a measure used to identify individuals who may be unable to complete unsupervised exercise safely or who need to consult a physician prior to exercising [18]. If the patient requires further approval prior to exercise, the RA will discuss this with the managing physician prior to initiating the Buffalo Concussion Treadmill Test (BCTT) in order to obtain medical clearance for exercise.

This study aims to enroll 30 WRC between June 2024 and May 2025. As a pilot study in an understudied population, determining nonadherence rates to THRAE is a key step in planning for future research trials. Previous studies from the CMCRC found nonadherence rates to THRAE between 10–30% in adolescent athletes and a loss of follow-up rate of 50% amongst concussed active duty military personnel [14,19]. Nonadherence rates among subjects with WRC are expected to be somewhere between these figures due to occupational differences (e.g., risk of military deployment) and potential differences in interest in and tolerance to exercise between athletes and injured workers. Therefore, given the 30-participant pool and a nonadherence rate between 10–50%, the precision of our nonadherence rate measured via the 95% confidence interval will have an estimated half width from between 9% to 15%. The upper limit of the confidence interval, combined with previously observed effect sizes [14,15], will allow computation of conservative sample size estimates for a future randomized controlled trial (RCT).

#### Inclusion criteria.

Adults 18–40 years old presenting within 6 weeks of work-related injury and diagnosed with concussion by an experienced sports medicine physician using standard criteria (described above). Potential participants must also be eligible for exercise based on completion of the PAR-Q + .

#### Exclusion criteria.

[1] 5-point or less difference between current and pre-injury symptoms as measured by the PCSS [2]; history of moderate or severe TBI [3]; current injury being more severe than mild, which includes loss of consciousness for >30 minutes, post-traumatic amnesia > 24 hours, focal neurological deficient on physical examination (or imaging if available) [4]; pre-existing conditions or presence of polytrauma that prevent participation in active testing and/or rehabilitation [5]; history of more than 3 diagnosed concussions [6]; active substance abuse/dependence; [7] report of injury mechanism occurring due to physical assault [8]; unwillingness to perform intervention; or [9] limited English proficiency.

Personal factors that may impact time to return to work and adherence to THRAE will be explored. These factors include the presence of depression, anxiety, symptoms of post-traumatic stress disorder (PTSD), as well as symptom burden, negative attitudes related to workplace climate, motivation, and psychological well-being. Each of these factors will be explored using validated patient-reported questionnaires, which will be administered after the patient provides consent to participate in the study and is cleared for exercise based on the PAR-Q+ measure (Table 1).

**Table 1:** Participant-reported questionnaires.

Measure Name	Domain Assessed
PCSS	Post-concussive symptom burden
Patient Health Questionnaire-9 (PHQ-9) [20]	Depression
Generalized Anxiety Disorder-7 (GAD-7) [21]	Anxiety
Primary Care PTSD Screen for DSM-5 (PC-PTSD-5) [22]	Factors suggestive of Post-Traumatic Stress Disorder
Patient-Reported Outcomes Measurement Information System Global Health-10 (PROMIS-10) [23]	Perceived physical, mental, and social health
Work Climate Questionnaire [24]	Motivation and psychological well-being focused on perceptions of autonomy and support received from workplace supervisors/ managers
Basic Psychological Needs Satisfaction and Frustration Scale (BPNSFS) [25]	Motivation and psychological well-being focused on satisfaction or frustration with basic psychological needs (i.e., autonomy, competence, relatedness)

**Fig 1:**
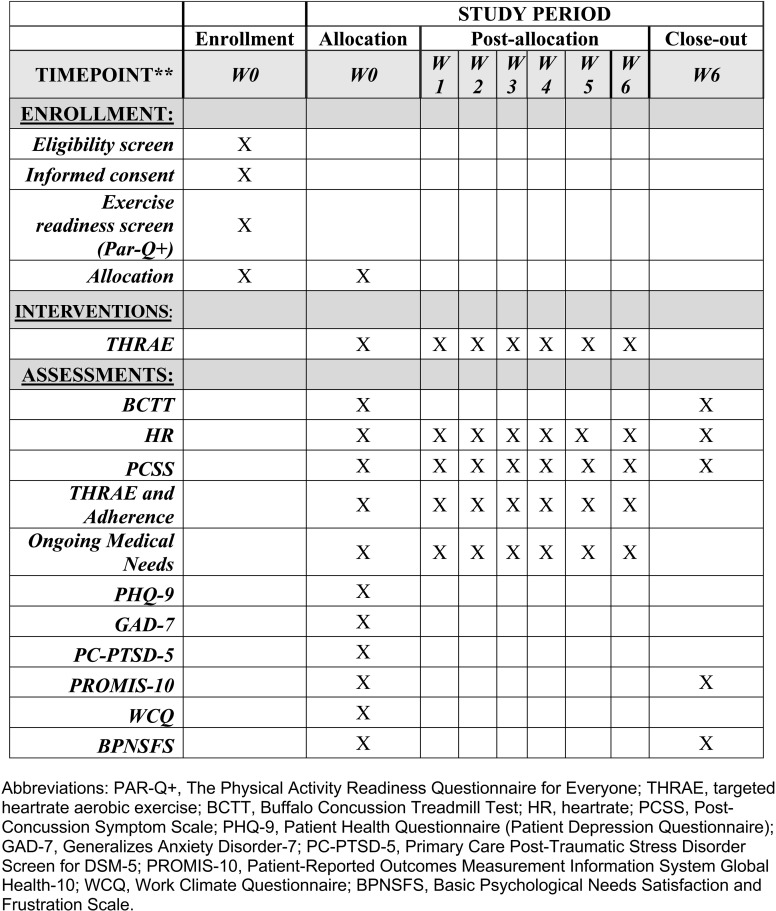
Schedule of Enrollment, Interventions, and Assessments. Abbreviations: PAR-Q + , The Physical Activity Readiness Questionnaire for Everyone; THRAE, targeted heartrate aerobic exercise; BCTT, Buffalo Concussion Treadmill Test; HR, heartrate; PCSS, Post-Concussion Symptom Scale; PHQ-9, Patient Health Questionnaire (Patient Depression Questionnaire); GAD-7, Generalizes Anxiety Disorder-7; PC-PTSD-5, Primary Care Post-Traumatic Stress Disorder Screen for DSM-5; PROMIS-10, Patient-Reported Outcomes Measurement Information System Global Health-10; WCQ, Work Climate Questionnaire; BPNSFS, Basic Psychological Needs Satisfaction and Frustration Scale.

An overview of the proposed study protocol is available in Fig 1. Following consent, exercise readiness verification via the PAR-Q + , and completion of the self-reported questionnaires, participants will be asked to complete the BCTT, a safe, graded exercise test used to identify concussion-related exercise intolerance [26]. Participants will wear a heart rate (HR) monitor to collect continuous HR data. The test is stopped when a participant’s symptoms increase subjectively by an intensity of 3 points or more from the pre-exercise value on a scale from 0–10, or they report being physically exhausted. Their HR at the time of test termination constitutes the HR threshold (HRt). An individualized THRAE training program will be prescribed based on 80% of the HRt on the BCTT [26].

Participants will be given a Polar HR monitor to wear while performing their THRAE prescription. They will be instructed to exercise at home for 20 minutes, 4–5 days per week, for up to 6 weeks or until they are medically cleared from their concussion during one of their weekly physician visits. Participants will retain their status as a clinic patient during and after the study. Participation in this study does not affect their clinical care. Participants’ exercise performance will be tracked utilizing Polar’s online coaching dashboard (Polar Flow Coach) and reviewed weekly by the study’s RA. Participants who are adherent will be sent a text message of encouragement for their compliance. Participants who are determined to be non-adherent to exercise protocols (missing 2 or more exercise sessions in one week) will be contacted by the RA to discuss potential barriers and solutions to enhance their exercise participation. The RA, who is also an athletic trainer, will be available by email and phone to address issues related to Polar HR device operation, enrollment into the Polar coaching dashboard, or THRAE performance. The RA will check-in with participants weekly following their physician visits to discuss exercise performance and ask about any adverse events (e.g., increased concussion-related symptoms, muscle soreness, fatigue, etc.). Any adverse events will be reported by project staff as required by institutional guidelines.

### Data management and statistical analyses

Data will be entered into a study database by the RA. Data will be made available to project staff via a secure cloud storage service. Patients will be de-identified in accordance with the Health Insurance Portability and Accountability Act of 1996 (HIPAA) Privacy Rule. Enrollment rate will be calculated using a proportion of the number of subjects consented compared to the number of eligible potential subjects. Adherence rates will be determined using two measures. The first measure will include a self-report that each participant will complete at each weekly follow-up visit with the RA. During these visits, participants will be asked to report the number of exercise sessions they have performed. Participants will be considered adherent if they complete 65% or more of their prescribed exercise sessions. The second measure will utilize data from the Polar coaching dashboard to determine the number of exercise sessions completed per week, the duration of each exercise session, and the mean, maximum, and minimum heart rates during each session. Determination of successful adherence based on Polar data will be based on the completion of 65% or more of their prescribed exercise sessions [29]. The rationale for considering adherence using multiple measures, subjective and objective, is that limited consensus exists around best practices for measuring and determining adherence to exercise-based interventions [27,28]. The 65% threshold has been reported in previous literature [29]. Despite training with the RA, injured workers may have less familiarity and more difficulty using the Polar equipment and application compared to the athletic and adolescent populations. Measuring adherence using multiple methods will enable piloting of multiple approaches which may be useful when planning subsequent larger-scale study protocols.

Univariate statistics will be performed to describe the sample. Estimates of the means and standard deviations for outcome variables, including symptom burden, time to recovery, time to return to work, and psychosocial measures, including those related to motivation and work climate, will be calculated. Means with standard deviation will be used to inform power analyses for a larger clinical trial. Participants will also be stratified based on whether they recovered during the intervention period and baseline demographics and clinical characteristics will be compared. Adherence rates will be determined based on self-report and THRAE data captured by the Polar app. Lastly, the rate of any adverse events will be described. All analyses will be performed on SPSS Version 29.

### Status and timeline

Recruitment and data collection have begun as of July 2024, and we have recruited a portion of our intended sample size. Initial data have not been analyzed. We expect to continue recruiting for the next several months with completion during the spring or summer of 2025. Once completed, data will be analyzed and submitted for publication in the form of one or more peer-reviewed manuscripts. Results will also be summarized at ClinicalTrials.gov.

## Discussion

This is one of the first attempts using THRAE to treat adults with WRC. The results will inform future studies in this area. There are limited data describing the impact of personal factors (e.g., motivation, workplace climate, etc.) on recovery from WRC and this study will provide some knowledge of these elements. Due to the novel nature of applying the proposed intervention and assessment items to individuals with WRC, we have elected to begin with a feasibility trial since adherence and safety data are a key priority. We believe that this initial study is a necessary step in the development of this line of research. The data obtained from this proposed study will inform future studies with greater methodological rigor, including randomized control trials, and will be used to apply for future funding proposals.

## Supporting information

S1 Data SPIRIT_checklist. (DOC)

S2 Data IRB protocol. (PDF)
