# Supplementary material for: Feasibility trial protocol assessing the use of aerobic exercise to promote recovery from work-related concussion
Source: PLoS One. 2025 Jun 12;20(6):e0325701. doi: 10.1371/journal.pone.0325701 (PMC12161564; doi:10.1371/journal.pone.0325701)
Supplement: S2 Data — (PDF) [file pone.0325701.s002.pdf]

University at Buffalo Institutional Review Board (UBIRB)
Office of Research Compliance | Clinical and Translational Research Center Room 5018
875 Ellicott St. | Buffalo, NY 14203
UB Federalwide Assurance ID#: FWA00008824

Complete Research Protocol (HRP-503)

Table of Contents

Template Instructions 2
1.0 Study Summary 4
2.0 Objectives* 5
3.0 Scientific Endpoints* 5
4.0 Background* 6
5.0 Study Design* 7
6.0 Study Intervention/Investigational Agent 7
7.0 Local Number of Subjects 8
8.0 Inclusion and Exclusion Criteria* 8
9.0 Vulnerable Populations* 10
10.0 Eligibility Screening* 11
11.0 Recruitment Methods 11
12.0 Procedures Involved* 12
13.0 Study Timelines* 14
14.0 Setting 15
15.0 Community-Based Participatory Research 15
16.0 Resources and Qualifications 16
17.0 Other Approvals 17
18.0 Provisions to Protect the Privacy Interests of Subjects 17
19.0 Data Management and Analysis* 18
20.0 Confidentiality* 19
A. Confidentiality of Study Data 19
B. Confidentiality of Study Specimens 20
21.0 Provisions to Monitor the Data to Ensure the Safety of Subjects* 20
22.0 Withdrawal of Subjects* 22
23.0 Risks to Subjects* 22
24.0 Potential Benefits to Subjects* 23
25.0 Compensation for Research-Related Injury 23
26.0 Economic Burden to Subjects 24
27.0 Compensation for Participation 24
28.0 Consent Process 25
29.0 Waiver or Alteration of Consent Process 29
30.0 Process to Document Consent 30
31.0 Multi-Site Research (Multisite/Multicenter Only)* 30
32.0 Banking Data or Specimens for Future Use* 32

Template Instructions

Sections that do not apply:

- *In several sections, the addition of checkboxes for* ***Not Applicable*** *have been added to the template as responses.*
  - *If an N/A checkbox is present, select the appropriate justification from the list.*
  - *If an N/A checkbox is not present, or if none of the existing checkboxes apply to your study, you must write in your own justification.*
- *In addition:*
  - *For research where the only study procedures are records/chart review: Sections 6, 21, 22, 24, 25, 26 and 27 do not apply.*
  - *For exempt research: Section 6 may not apply. Section 6.1 will still apply if there is a study intervention.*

Studies with multiple participant groups:

- *If this study involves multiple participant groups (e.g. parents and children), provide information in applicable sections for each participant group. Clearly label responses when they differ. For example:*

  Response Example

  Intervention Group:

  Control Group:

Formatting:

- *Do not remove template instructions or section headings when they do not apply to your study.*

If you are pasting information from other documents using the "Merge Formatting" Paste option will maintain the formatting of the response boxes.

Amendments:

- *When making modifications or revisions to this and other documents, use the* ***Track Changes*** *function in Microsoft Word.*
- *Update the version date or number* ***on Page 3.***

PROTOCOL TITLE:

Include the full protocol title.

Response:

Feasibility of Aerobic Exercise for Recovery from Work-related Concussion

PRINCIPAL INVESTIGATOR:

Name
Department
Telephone Number
Email Address

Response:

Jacob I McPherson

Rehabilitation Sciences

716-829-6734

jim6@buffalo.edu

VERSION NUMBER/DATE:

Include the version number and date of this protocol.

Response:

1.0

12.05.23

REVISION HISTORY

Revision #	Version Date	Summary of Changes	Consent Change?

FUNDING:

Indicate any funding for this proposal. This should match the Funding Sources page in Click IRB.

Response:

Approved for CTSI Seed grant funding. Grant number: PS-MCPHERSON under UL1TR001412

GRANT APPLICABILITY:

Indicate whether this protocol is funded by a grant (e.g. NIH, foundation grant). For a grant with multiple aims, indicate which aims are covered by this research proposal.

NOTE: This question does not apply to studies funded by a sponsor contract.

Include a copy of the grant proposal with your submission.

Response:

CTSI Seed grant funding. Grant number: PS-MCPHERSON under UL1TR001412

RESEARCH REPOSITORY:

Indicate where the research files will be kept, including when the study has been closed. The repository should include, at minimum, copies of IRB correspondence (approval, determination letters) as well as signed consent documents. This documentation should be maintained for 3 years after the study has been closed.

Response:

Location: Office of Dr McPherson

Address: 534 Kimball, Buffalo, NY, 14214

Department: Rehabilitation Science

1.0 Study Summary

**Study Title**	Feasibility of Aerobic Exercise for Recovery from Work-related Concussion
**Study Design**	Prospective cohort
**Primary Objective**	Identify the feasibility of implementing a targeted heart rate aerobic exercise (THRAE) intervention in patients with work-related concussion<br>Identify what practical adjustments are necessary when implementing THRAE to a non-athlete population
**Secondary Objective(s)**	Identify adherence rates to THRAE and incidence of adverse events
**Research Intervention(s)/ Investigational Agent(s)**	THRAE (a mild to moderate exercise prescription that has been validated to be effective in the athlete population)
**IND/IDE #**	NA
**Study Population**	Adults with work-related concussions
**Sample Size**	40
**Study Duration for individual participants**	Up to 6 weeks

**Study Specific Abbreviations/ Definitions**	WRC – work related concussion<br>SRC – sport related concussion<br>THRAE – targeted heart rate aerobic exercise<br>PPCS – persistent post-concussive symptoms

2.0 Objectives*

2.1 Describe the purpose, specific aims, or objectives of this research.

Response: We propose a prospective feasibility trial to evaluate a proven treatment approach, target heart rate aerobic exercise (THRAE), to sport related to concussion (SRC) applied to individuals with work-related concussion (WRC), an understudied population. This project will provide data critical to the development of a federally funded randomized controlled trial. Key elements of interest from this trial include:

1. Estimates of the means and standard deviations for outcome variables, including symptom burden, time to recovery, time to return to work, and psychosocial measures related to motivation and work climate.
2. Adherence rates to THRAE within a newly studied patient group (patients with WRC).
3. Incidence of adverse events.
4. Practical adjustments to existing SRC THRAE protocols necessary for application to WRC patients.

2.2 State the hypotheses to be tested, if applicable.

NOTE: A hypothesis is a specific, testable prediction about what you expect to happen in your study that corresponds with your above listed objectives.

Response: N/A as this is a feasibility trial.

3.0 Scientific Endpoints*

3.1 Describe the scientific endpoint(s), the main result or occurrence under study.

NOTE: Scientific endpoints are outcomes defined before the study begins to determine whether the objectives of the study have been met and to draw conclusions from the data. Include primary and secondary endpoints. Some example endpoints are: reduction of symptoms, improvement in quality of life, or survival. Your response should ***not*** *be a date.*

Response:

Estimates of the means and standard deviations for outcome variables, including symptom burden, time to recovery, time to return to work, psychosocial measures related to motivation and work climate and adherence rates

4.0 Background*

4.1 Provide the scientific or scholarly background, rationale, and significance of the research based on the existing literature and how it will contribute to existing knowledge. Describe any gaps in current knowledge. Include relevant preliminary findings or prior research by the investigator.

Response:

According to the Occupational Health and Safety Administration and the Centers for Disease Control, the average direct cost of a single work-related concussion (WRC) is nearly $55,000, (1) resulting in an annual U.S. cost of $1.2 billion. By contrast, the cost of care for a sport-related concussion (SRC) is estimated to be $800-$3,500. (2) Significantly less research and media attention have been directed at WRC. The early active rehabilitation program implemented within 2 weeks after SRC developed at UB, which utilizes targeted heart rate aerobic exercise (THRAE), has transformed concussion treatment internationally. (3,4) Evidence-based treatments for individuals with WRC are limited, and while the literature has identified unique demographic and psychosocial factors inherent to this population (e.g., socioeconomic status and financial strain, occupation, motivation, life roles, and cognitive status), (5,6) they are often expected to recover like athletes with SRC.

Our pilot data, however, have demonstrated that adults with WRC take more than 8x longer to recover compared to individuals with SRC. Evidence suggests that the longer an injured worker is out of work, the less likely they are to rejoin the workforce.(5) Not only does this have tremendous financial implications for workers and their families, but maintaining an adequate workforce is critical these days. Furthermore, Black/African Americans and Hispanic/Latino groups experience a disproportionate rate of work-related injuries and illnesses; a phenomenon that requires further investigation.

4.2 Include complete citations or references.

Response:

1. Occupational Safety and Health Administration. Estimated costs of occupational injuries and illnesses and estimated impact on a company's profitability Worksheet. Washington, DC. 2010.

2. Yengo-Kahn AM, Kelly PD, Liles DC, McKeithan LJ, Grisham CJ, Khan MS, Lee T, Kuhn AW, Bonfield CM, Zuckerman SL. The cost of a single concussion in American high school football: a retrospective cohort study. Concussion. 2020 Dec;5(4):CNC81.

3. Leddy JJ, Haider MN, Ellis MJ, Mannix R, Darling SR, Freitas MS, Suffoletto HN, Leiter J, Cordingley DM, Willer B. Early subthreshold aerobic exercise for sport-related concussion: a randomized clinical trial. JAMA pediatrics. 2019 Apr 1;173(4):319-25.

4. Leddy JJ, Master CL, Mannix R, Wiebe DJ, Grady MF, Meehan WP, Storey EP, Vernau BT, Brown NJ, Hunt D, Mohammed F, Mallon A, Rownd K, Arbogast KA, Cunningham A, Haider MN, Mayer AR, Willer BS. Early targeted heart rate aerobic exercise versus placebo stretching for sport-related concussion in adolescents: a randomised controlled trial. The Lancet Child & Adolescent Health. 2021 Nov 1;5(11):792-9.

5. Thompson A, Chechulin Y, Bain D, Bayley M. An evidence-based care model for workers with concussion. The Journal of Head Trauma Rehabilitation. 2019 Sep 1;34(5):E10-6.

6. Slavova S, Bunn TL. Work-related concussion surveillance. American journal of industrial medicine. 2015 Jan;58(1):40-5.

5.0 Study Design*

5.1 Describe and explain the study design (e.g. case-control, cross-sectional, ethnographic, experimental, interventional, longitudinal, observational).

Response:

Prospective cohort with intervention

6.0 Study Intervention/Investigational Agent

6.1 Describe the study intervention and/or investigational agent (e.g., drug, device) that is being evaluated.

Response:

The experimental intervention is a prescription for THRAE. It involves performing a 15-20 minute graded exercise tolerance test on a treadmill at the clinic, and performing around 20 minutes of aerobic exercise (i.e. walking, jogging) 4-5 days per week..

6.2 Drug/Device Handling: If the research involves drugs or device, describe your plans to store, handle, and administer those drugs or devices so that they will be used only on subjects and be used only by authorized investigators.

- *If the control of the drugs or devices used in this protocol will be accomplished by following an established, approved organizational SOP (e.g., Research Pharmacy SOP for the Control of Investigational Drugs, etc.), please reference that SOP in this section.*

Response:

N/A

6.3 If the drug is investigational (has an IND) or the device has an IDE or a claim of abbreviated IDE (non-significant risk device), include the following information:

- *Identify the holder of the IND/IDE/Abbreviated IDE.*
- *Explain procedures followed to comply with sponsor requirements for FDA regulated research for the following:*

	Applicable to:		
FDA Regulation	***IND Studies***	***IDE studies***	***Abbreviated IDE studies***
21 CFR 11	***X***	***X***	
21 CFR 54	***X***	***X***	
21 CFR 210	***X***		
21 CFR 211	***X***		
21 CFR 312	***X***		
21 CFR 812		***X***	***X***
21 CFR 820		***X***	

Response:

N/A

7.0 Local Number of Subjects

7.1 Indicate the total number of subjects that will be enrolled or records that will be reviewed locally.

Response:

40

7.2 If applicable, indicate how many subjects you expect to screen to reach your target sample (i.e. your screen failure rate).

Response:

60

7.3 Justify the feasibility of recruiting the proposed number of eligible subjects within the anticipated recruitment period. For example, how many potential subjects do you have access to? What percentage of those potential subjects do you need to recruit?

Response:

The UBMD concussion clinic sees approximately 50 patients with a concussion every week, and about 30% of them are injured workers with WRC (the remainder being sport-related). Our research assistant will be present during clinic hours and will be able to consent prospective participants on the same date of their initial medical evaluation.

8.0 Inclusion and Exclusion Criteria*

8.1 Describe the criteria that define who will be ***included*** *in your final study sample.*

NOTE: This may be done in bullet point fashion.

Response:

- Aged 18-40
- Received a concussion at work and are engaged with the workers compensation program
- Within 3 weeks of concussive injury

8.2 *Describe the criteria that define who will be **excluded** from your final study sample.*

NOTE: This may be done in bullet point fashion.

Response:

(1) 3-point or less difference between current and pre-injury symptoms as measured by the Post-Concussion Symptom Inventory (PCSI); (2) moderate or severe TBI; (3) injury involving loss of consciousness for >30 minutes or post-traumatic amnesia > 24 hours; (4) pre-existing conditions or presence of polytrauma that prevent participation in active testing and/or rehabilitation; (5) history of more than 3 diagnosed concussions; (6) active substance abuse/dependence; (7) report of injury mechanism occurring due to physical assault; 8) unwillingness to perform intervention; or (9) limited English proficiency.

8.3 *Indicate specifically whether you will include any of the following special populations in your study using the checkboxes below.*

NOTE: Members of special populations may not be targeted for enrollment in your study unless you indicate this in your inclusion criteria.

Response:

☐ Adults unable to consent

☐ Individuals who are not yet adults (infants, children, teenagers)

☐ Pregnant women

☐ Prisoners

8.4 *Indicate whether you will include non-English speaking individuals in your study.* ***Provide justification if you will exclude non-English speaking individuals.***

*In order to meet one of the primary ethical principles of equitable selection of subjects, non-English speaking individuals may **not** be routinely excluded from research as a matter of convenience.*

In cases where the research is of therapeutic intent or is designed to investigate areas that would necessarily require certain populations who may not speak English, the researcher is required to make efforts to recruit and include non-English speaking individuals. However, there are studies

in which it would be reasonable to limit subjects to those who speak English. Some examples include pilot studies, small unfunded studies with validated instruments not available in other languages, studies with numerous questionnaires, and some non-therapeutic studies which offer no direct benefit.

Response:

We will not be including non-English speaking participants. The UBMD Concussion Clinic has limited translating services for clinicians and the athletic trainers who perform the exercise test do not have any translating services available. This is a small-scale feasibility trial without the resources necessary to secure translation services.

9.0 Vulnerable Populations*

If the research involves special populations that are considered vulnerable, ***describe the safeguards included to protect their rights and welfare.***

NOTE: You should refer to the appropriate checklists, referenced below, to ensure you have provided adequate detail regarding safeguards and protections. You do not, however, need to provide these checklists to the IRB.

9.1 For research that involves ***pregnant women,*** *safeguards include:*
NOTE CHECKLIST: Pregnant Women (HRP-412)

Response:

☒ **N/A**: This research does not involve pregnant women.

9.2 For research that involves ***neonates of uncertain viability or non-viable neonates,*** *safeguards include:*
NOTE CHECKLISTS: Non-Viable Neonates (HRP-413), or Neonates of Uncertain Viability (HRP-414)

Response:

☒ **N/A:** This research does not involve non-viable neonates or neonates of uncertain viability.

9.3 For research that involves ***prisoners****, safeguards include:*
NOTE CHECKLIST: Prisoners (HRP-415)

Response:

☒ **N/A:** This research does not involve prisoners.

9.4 For research that involves ***persons who have not attained the legal age for consent to treatments or procedures involved in the research ("children")****, safeguards include:*
NOTE CHECKLIST: Children (HRP-416)

Response:

☒ **N/A:** This research does not involve persons who have not attained the legal age for consent to treatments or procedures ("children").

*9.5 For research that involves **cognitively impaired adults**, safeguards include:*
NOTE CHECKLIST: Cognitively Impaired Adults (HRP-417)

Response:

☒ **N/A:** This research does not involve cognitively impaired adults.

*9.6 Consider if other specifically targeted populations such as students, employees of a specific firm, or educationally or economically disadvantaged persons are vulnerable. **Provide information regarding their safeguards and protections, including safeguards to eliminate coercion or undue influence.***

Response:

No additional vulnerable populations are identified.

10.0 Eligibility Screening*

*10.1 Describe **screening procedures** for determining subjects' eligibility.*
Screening refers to determining if prospective participants meet inclusion and exclusion criteria.

Include all relevant screening documents with your submission (e.g. screening protocol, script, questionnaire).

Response:

The study physician will diagnose the concussion and see if they are eligible to participate in the study (age range and days since injury). If eligible, then the research assistant will use the Physical Activity Readiness- Questionnaire (PAR-Q) to screen for contraindications to exercise. The PAR-Q is attached.

☐ **N/A:** There is no screening as part of this protocol.

11.0 Recruitment Methods

☐ **N/A:** This is a records review only, and subjects will not be recruited. NOTE: If you select this option, please make sure that all records review procedures and inclusion/exclusion screening are adequately described in other sections.

11.1 Describe when, where, and how potential subjects will be recruited.

NOTE: Recruitment refers to how you are identifying potential participants and introducing them to the study. Include specific methods you will use (e.g. searching charts for specific ICD code numbers, Research Participant Groups, posted advertisements, etc.).

Response:

Prospective participants will come to the UBMD Concussion Clinic to be seen for their concussion injury. The UBMD Concussion Clinic is well-known in the area and is a direct referral site for occupational injury triage facilities. The treating physician, who is also a member of the research team, will perform a clinician examination in private exam rooms and diagnose them with a concussion or not. If diagnosed with a concussion and eligible to participate, the study physician will inform them about the study and ask a research assistant to come to the exam room and explain the study and obtain consent (if interested).

11.2 Describe how you will protect the privacy interests of prospective subjects during the recruitment process.

NOTE: Privacy refers to an individual's right to control access to him or herself.

Response:

Prospective participants will be informed that participation is voluntary and not participating will not alter their clinical care. All discussions during the recruitment process will take place in a private exam room.

11.3 Identify any materials that will be used to recruit subjects.

NOTE: Examples include scripts for telephone calls, in person announcements / presentations, email invitations.

For advertisements, include the final copy of printed advertisements with your submission. When advertisements are taped for broadcast, attach the final audio/video tape. NOTE: You may submit the wording of the advertisement prior to taping to ensure there will be no IRB-required revisions, provided the IRB also reviews and approves the final version.

Response:

None

12.0 Procedures Involved*

*12.1 Provide a description of **all research procedures or activities** being performed and when they are performed once a subject is screened and determined to be eligible. Provide as much detail as possible.*

NOTE: This should serve as a blueprint for your study and include enough detail so that another investigator could pick up your protocol and replicate the research. For studies that have multiple or complex visits or procedures, consider the addition of a schedule of events table in in your response.

Response:

Participants who are diagnosed with a concussion and agree to participant in the study will complete a demographics form and other relevant questionnaires. They will then perform a graded exertion test (the Buffalo Concussion Treadmill Test [BCTT]) at the clinic, this takes approximately 15 minutes. Participants will then be prescribed the individualized THRAE based on the results from the BCTT and will be sent home with a commercial heart rate monitor. Participants will perform exercises at home on their free time 4-5 days per week. Participants will return to

the clinic weekly to be re-examined by the study physician and obtain a new heart rate prescription for the first six weeks until clinical recovery or the intervention period ends (6-weeks).

12.2 Describe what data will be collected.

NOTE: For studies with multiple data collection points or long-term follow up, consider the addition of a schedule or table in your response.

Response:

Demographics at initial visit

- Patient last name and date of visit (identifiable data)
- Contact information (email, phone, zip code)
- Age, Sex, race, student/employment status, occupation
- Height, weight
- Days since injury
- Method of injury
- Work and concussion history
- Past medical and medication history

Patient reported measures

- Concussion symptoms checklist (PCSI)
- PHQ-9, GAD-7, PTSD-5, PROMIS-10
- Work Climate Questionnaire, BPNSFN

Physical assessment (Buffalo Concussion Physical Exam [BCPE])

- Cervical assessment results
- Oculomotor assessment results
- Balance assessment results

The Physical Activity Readiness Questionnaire for Everyone (PAR-Q+)

Exercise

- Level of exercise tolerance (i.e. the heart rate threshold obtained from the BCTT)
- Symptoms exacerbated and rating of perceived exertion on BCTT

Follow-up questionnaire

- Self-reported level of recovery
- Adherence rate to prescribed exercise
- Incidence of adverse events
- Date of recovery (if applicable)

12.3 List any instruments or measurement tools used to collect data (e.g. questionnaire, interview guide, validated instrument, data collection form).

Include copies of these documents with your submission.

Response:

The demographics, symptom checklist (PCSS), BCTT sheet, PHQ-9, GAD-7, PC-PTSD-5, PROMIS-10, Work Climate Questionnaire, BPNSFN are attached as "Study Booklet."

12.4 Describe any source records that will be used to collect data about subjects (e.g. school records, electronic medical records).

Response:

Electronic medical records of the UBMD Concussion Clinic will be used to obtain the physician's examination findings. The UBMD Concussion Clinic utilizes Medent as their electronic medical record. We will obtain medical records for study participants from Medent.

12.5 Indicate whether or not ***individual*** *subject results, such as results of investigational diagnostic tests, genetic tests, or incidental findings will be shared with subjects or others (e.g., the subject's primary care physician) and if so, describe how these will be shared.*

Response:

Results of exercise tolerance test will be shared with the participant immediately when it is completed.

12.6 Indicate whether or not ***study*** *results will be shared with subjects or others, and if so, describe how these will be shared.*

Response:

We will aim to published grouped result in peer-reviewed medical journals.

13.0 Study Timelines*

13.1 Describe the anticipated duration needed to enroll all study subjects.

Response:

12 months

13.2 Describe the duration of an individual subject's participation in the study. Include length of study visits, and overall study follow-up time.

Response:

Approximately 10-15 minutes extra at each weekly clinic visit for up to 6-weeks. Independent exercise for 20 minutes occurring 4-5 days per week.

13.3 Describe the estimated duration for the investigators to complete this study (i.e. all data is collected and all analyses have been completed).

Response:

18 months

14.0 Setting

14.1 Describe all facilities/sites where you will be conducting research procedures. Include a description of the security and privacy of the facilities (e.g. locked facility, limited access, privacy barriers). Facility, department, and type of room are relevant. Do not abbreviate facility names.

NOTE: Examples of acceptable response may be: "A classroom setting in the Department of Psychology equipped with a computer with relevant survey administration software," "The angiogram suite at Buffalo General Medical Center, a fully accredited tertiary care institution within New York State with badge access," or, "Community Center meeting hall."

Response:

The UBMD Concussion Management Clinic is a standard outpatient medical clinic setting with private exam rooms and exercise testing facilities (i.e. a room with a treadmill) and follows all patient confidentiality practices.

14.2 For research conducted outside of UB and its affiliates, describe:

- *Site-specific regulations or customs affecting the research*
- *Local scientific and ethical review structure*

NOTE: This question is referring to UB affiliated research taking place outside UB, i.e. research conducted in the community, school-based research, international research, etc. It is not referring to multi-site research. UB affiliated institutions include Kaleida Health, ECMC, and Roswell Park Cancer Institute.

Response:

☒ **N/A:** This study is not conducted outside of UB or its affiliates.

15.0 Community-Based Participatory Research

15.1 Describe involvement of the community in the design and conduct of the research.

NOTE: Community-Based Participatory Research (CBPR) is a collaborative approach to research that equitably involves all partners in the research process and recognizes the unique strengths that each brings. CBPR begins with a research topic of importance to the community, has the aim of combining knowledge with action and achieving social change to improve health outcomes and eliminate health disparities.

Response:

☒ **N/A:** This study does not utilize CBPR.

15.2 Describe the composition and involvement of a community advisory board.

Response:

☒ **N/A:** This study does not have a community advisory board.

16.0 Resources and Qualifications

16.1 Describe the qualifications (e.g., education, training, experience, expertise, or certifications) of the Principal Investigator ***and*** *staff to perform the research. When applicable describe their knowledge of the local study sites, culture, and society. Provide enough information to convince the IRB that you have qualified staff for the proposed research.*

NOTE: If you specify a person by name, a change to that person will require prior approval by the IRB. If you specify a person by role (e.g., coordinator, research assistant, co-investigator, or pharmacist), a change to that person will not usually require prior approval by the IRB, provided that the person meets the qualifications described to fulfill their roles.

Response:

Jacob I McPherson, DPT, PhD – Clinical Assistant Professor, Department of Rehabilitation Sciences, is experienced with the clinical management of concussion and providing physical rehabilitation.

Chris Stavisky, PhD, OTR/L – Clinical Assistant Professor, Department of Rehabilitation Sciences, is experienced with the clinical management of concussion and providing occupational rehabilitation.

M Nadir Haider, MD, PhD – Research Assistant Professor, Department of Orthopedics, is the assistant director of the UBMD Concussion Clinic and experienced with treatment research

Haley M Chizuk, ATC, PhD – Post-Doctoral Associate Department of Orthopedics, is experienced with prescribing exercise to patients with concussion

Jeff C Miecznikowski, PhD – Associate Professor, Department of Biostatistics, is an expert in statistical analysis

John J Leddy, MD – Clinical Professor, Department of Orthopedics, is the director of the UBMD Concussion Clinic, a world-renowned concussion specialist, and the one to developed the THRAE

Describe other resources available to conduct the research.

16.2 Describe the time and effort that the Principal Investigator and research staff will devote to conducting and completing the research.

NOTE: Examples include the percentage of Full Time Equivalents (FTE), hours per week. The question will elicit whether there are appropriate resources to conduct the research.

Response:

McPherson – 20%

Stavisky – 20%

Haider – 5%

Chizuk – 15%

Miecznikowski – 5%

Leddy – 5%

16.3 Describe the availability of medical or psychological resources that subjects might need as a result of anticipated consequences of the human research, if applicable.

NOTE: One example includes: on-call availability of a counselor or psychologist for a study that screens subjects for depression.

Response:

The UBMD Concussion Management Clinic is within the UBMD Orthopedics and Sports Medicine healthcare facility and always has physicians, surgeons, psychiatrists, psychologists, and therapists available for treatment.

16.4 Describe your process to ensure that all persons assisting with the research are adequately informed about the protocol, the research procedures, and their duties and functions.

Response:

Weekly research meetings will be held throughout the enrollment period to ensure everyone is following protocol and review any problems (if applicable).

17.0 Other Approvals

17.1 Describe any approvals that will be obtained prior to commencing the research (e.g., school, external site, funding agency, laboratory, radiation safety, or biosafety).

Response:

☒ **N/A:** This study does not require any other approvals.

18.0 Provisions to Protect the Privacy Interests of Subjects

18.1 Describe how you will protect subjects' privacy interests during the course of this research.

NOTE: Privacy refers to an individual's right to control access to him or herself. Privacy applies to the person. Confidentiality refers to how data collected about

individuals for the research will be protected by the researcher from release. Confidentiality applies to the data.

Examples of appropriate responses include: "participant only meets with a study coordinator in a classroom setting where no one can overhear", or "the participant is reminded that they are free to refuse to answer any questions that they do not feel comfortable answering."

Response:

Participants will be told they can end their participation within the study whenever they choose and can resume standard care. Study-related office visits only include the participant and research staff members in a private room.

18.2 Indicate how the research team is permitted to access any sources of information about the subjects.

*NOTE: Examples of appropriate responses include: school permission for review of records, consent of the subject, HIPAA waiver. This question **does apply** to records reviews.*

Response:

Written consent from the participant.

19.0 Data Management and Analysis*

19.1 Describe the data analysis plan, including any statistical procedures. This section applies to both quantitative and qualitative analysis.

Response:

Univariate statistics will be performed to describe the sample. Estimates of the means and standard deviations for outcome variables, including symptom burden, time to recovery, time to return to work, and psychosocial measures related to motivation and work climate will be calculated. Participants will then be stratified if they recovered or not during the intervention period and baseline demographics and clinical characteristics will be compared. Lastly, the rate of adverse events (if any) will be described. All analysis will be performed on SPSS Version 29.

19.2 If applicable, provide a power analysis.

NOTE: This may not apply to certain types of studies, including chart/records reviews, survey studies, or observational studies. This question is asked to elicit whether the investigator has an adequate sample size to achieve the study objectives and justify a conclusion.

Response:

No a priori sample size estimation can be performed because there is no published or pilot data about implementing the THRAE in patients with WRC.

19.3 Describe any procedures that will be used for quality control of collected data.

Response:

Drs. McPherson and Stavisky will review an individual's data collected when they have completed their intervention to screen for errors and missing data.

20.0 Confidentiality*

A. Confidentiality of Study Data

*Describe the local procedures for maintenance of confidentiality of **study data and any records that will be reviewed for data collection**.*

*20.1 A. Where and how will all data and records be stored? Include information about: password protection, encryption, physical controls, authorization of access, and separation of identifiers and data, as applicable. Include physical (e.g. paper) **and** electronic files.*

Response:

Clinical data (symptom questionnaire, physical assessment and BCTT) will be stored in the patient's electronic medical record (Medent) directly and will be retrospectively extracted. These EMRs are secure and HIPAA-compliant.

Consent forms, demographics and follow-up questionnaires will be collected in paper forms and stored at the office of Dr Jacob McPherson.

20.2 A. How long will the data be stored?

Response:

Identifiable data (patient last name and date of clinic visit) will be deleted as soon as the study is complete. It is only required to extract medical information from EMR. A limited data set (including PHI identifier of dates of service) will be stored for a minimum of 10 years.

20.3 A. Who will have access to the data?

Response:

Members of the research team

20.4 A. Who is responsible for receipt or transmission of the data?

Response:

Dr McPherson

20.5 A. How will the data be transported?

Response:

The identifiable list of patient names and study ID key will be stored on UB Box, which is a secure, university-managed server. Research members will access this list online to update the list when a new participant is added.

Paper files (which has de-identified data only) will be transported by hand from the Concussion Clinic to the Office of Dr McPherson.

B. Confidentiality of Study Specimens

*Describe the local procedures for maintenance of confidentiality of **study specimens**.*

☒ **N/A:** No specimens will be collected or analyzed in this research.
(Skip to Section 21.0)

20.6 B. Where and how will all specimens be stored? Include information about: physical controls, authorization of access, and labeling of specimens, as applicable.

Response:

20.7 B. How long will the specimens be stored?

Response:

20.8 B. Who will have access to the specimens?

Response:

20.9 B. Who is responsible for receipt or transmission of the specimens?

Response:

20.10 B. How will the specimens be transported?

Response:

21.0 Provisions to Monitor the Data to Ensure the Safety of Subjects*

☐ **N/A:** This study is not enrolling subjects, or is limited to records review procedures only. This section does not apply.

NOTE: Minimal risk studies may be required to monitor subject safety if the research procedures include procedures that present unique risks to subjects that require monitoring. Some examples include: exercising to exertion, or instruments that elicit suicidality or substance abuse behavior. In such cases, N/A is not an acceptable response.

21.1 Describe the plan to periodically evaluate the data collected regarding both harms and benefits to determine whether subjects remain safe.

Response:

Participants will be asked at every follow-up appointment if they had any problems with the home exercise prescription and if they were any adverse events (i.e. injuries).

21.2 Describe what data are reviewed, including safety data, untoward events, and efficacy data.

Response:

Incidence of injuries or near missed while performing exercises at home.

21.3 Describe any safety endpoints.

Response:

If a patient is injured while exercising at home, then they will be seen by the study physician to see if the injury will affect their concussion recovery and ability to perform exercise at home. If the injury is more than just a mild inconvenience (e.g., post-exercise soreness), then the study intervention will be stopped.

21.4 Describe how the safety information will be collected (e.g., with case report forms, at study visits, by telephone calls with participants).

Response:

At follow-up visits using a standardized follow-up form.

21.5 Describe the frequency of safety data collection.

Response:

Weekly

21.6 Describe who will review the safety data.

Response:

Dr Leddy, who is a board-certified sports medicine physician.

21.7 Describe the frequency or periodicity of review of cumulative safety data.

Response:

Cumulative safety data will be reviewed once a month.

21.8 Describe the statistical tests for analyzing the safety data to determine whether harm is occurring.

Response:

No analysis plan is identified in the prospective sample of 40 participants. We have conducted several trials in the past using THRAE without ever having an adverse event or a near-miss.

21.9 Describe any conditions that trigger an immediate suspension of the research.

Response:

If there is any indication that performing the THRAE worsens concussion recovery, then the study will be suspended.

22.0 Withdrawal of Subjects*

☐ **N/A:** This study is not enrolling subjects. This section does not apply.

*22.1 Describe **anticipated** circumstances under which subjects may be withdrawn from the research without their consent.*

Response:

Participants who do not return to the clinic or are unwilling to perform exercise after they have agreed to perform it will be removed.

22.2 Describe any procedures for orderly termination.

NOTE: Examples may include return of study drug, exit interview with clinician. Include whether additional follow up is recommended for safety reasons for physical or emotional health.

Response:

Participants will be asked to end their experimental intervention, return the heart rate monitor, and return to the clinic according their clinic needs as determined by their managing physician.

22.3 Describe procedures that will be followed when subjects withdraw from the research, including retention of already collected data, and partial withdrawal from procedures with continued data collection, as applicable.

Response: Already collected data will be retained for later analyses to determine factors related to study withdrawal.

23.0 Risks to Subjects*

23.1 List the reasonably foreseeable risks, discomforts, hazards, or inconveniences to the subjects related to their participation in the research. Consider physical, psychological, social, legal, and economic risks. Include a description of the probability, magnitude, duration, and reversibility of the risks.

NOTE: Breach of confidentiality is always a risk for identifiable subject data.

Response:

Graded exertion testing makes people feel tired and out of breath, it may mildly worsen concussion-like symptoms in patients who are exercise intolerant, which

makes the test positive. This is the expected response and is not considered an adverse event.

Breach of confidentiality is a risk.

23.2 Describe procedures performed to lessen the probability or magnitude of risks, including procedures being performed to monitor subjects for safety.

Response:

Graded exertion testing is performed by athletic trainers, the test ends when patients become symptomatic. These symptoms tend to reside after 10 minutes of rest.

The risk of breach in confidentiality will be mitigated by keeping the study ID key (that has identifiable information) separate from the paper data collection forms.

23.3 If applicable, indicate ***which procedures*** *may have risks to the subjects that are currently unforeseeable.*

Response:

None are identified.

23.4 If applicable, indicate which research procedures may have risks to an embryo or fetus should the subject be or become pregnant.

Response:

None are identified.

23.5 If applicable, describe risks to others who are not subjects.

Response:

None are identified.

24.0 Potential Benefits to Subjects*

24.1 Describe the potential benefits that individual subjects may experience by taking part in the research. Include the probability, magnitude, and duration of the potential benefits. Indicate if there is no direct benefit.

NOTE: Compensation ***cannot*** *be stated as a benefit.*

Response:

Participants may recover and return to asymptomatic status sooner if they participate in the research. This has been proven for SRC and the purpose of this study is to see if it will also have an effect on WRC.

25.0 Compensation for Research-Related Injury

☐ **N/A:** The research procedures for this study do not present risk of research related injury (e.g. survey studies, records review studies). This section does not apply.

*25.1 **If the research procedures carry a risk of research related injury,** describe the available compensation to subjects in the event that such injury should occur.*

Response:

There is no available compensation for research related injury.

25.2 Provide a copy of contract language, if any, relevant to compensation for research related injury.

*NOTE: If the contract is not yet approved at the time of this submission, submit the current version here. If the contract is later approved with **different language regarding research related injury**, you must modify your response here and submit an amendment to the IRB for review and approval.*

Response:

It is important that you tell your study doctor if you feel that taking part in this study has injured you or caused you to become ill.

You will receive medical treatment if you are injured or become ill as a result of this study. Your doctor will explain the treatment options to you and tell you where you can get treatment.

The University at Buffalo makes no commitment to provide free medical care or payment for any unfavorable outcomes that may result from your participation in this research. Medical services will be billed at the usual chart and will be your responsibility or that of your third-party payer, but you are not precluded from seeking to collect compensation for injury related to malpractice, fault, or blame on the part of those involved in the research including the University at Buffalo.

26.0 Economic Burden to Subjects

26.1 Describe any costs that subjects may be responsible for because of participation in the research.

NOTE: Some examples include transportation or parking.

Response:

Participants will need to make time to exercise at home. The weekly clinic visits, including transportation and parking, are part of standard care so it should not be added to the research cost.

☐ **N/A:** This study is not enrolling subjects, or is limited to records review procedures only. This section does not apply.

27.0 Compensation for Participation

27.1 Describe the amount and timing of any compensation to subjects, including monetary, course credit, or gift card compensation.

Response:

Participants will be paid $30 per visit for their initial visit and each follow-up visit (up to 5 total visits) plus $100 for their final visit if they have not missed a

previous appointment. Payments will be made at the time of the initial visit and at each follow-up visit. The maximum payment per study participant is $250. All payments will be made using preloaded subject payment debit cards.

☐ **N/A:** This study is not enrolling subjects, or is limited to records review procedures only. This section does not apply.

☐ **N/A:** There is no compensation for participation. This section does not apply.

28.0 Consent Process

28.1 Indicate whether you will be obtaining consent.

NOTE: This does not refer to consent documentation, but rather whether you will be obtaining permission from subjects to participate in a research study. Consent documentation is addressed in Section 29.0.

☒ ***Yes*** *(If yes, Provide responses to each question in this Section)*
☐ ***No*** *(If no, Skip to Section 29.0)*

28.2 Describe where the consent process will take place. Include steps to maximize subjects' privacy.

Response:

Consent will take place in private medical exam rooms.

28.3 Describe how you will ensure that subjects are provided with a sufficient period of time to consider taking part in the research study.

NOTE: It is always a requirement that a prospective subject is given sufficient time to have their questions answered and consider their participation. See "SOP: Informed Consent Process for Research (HRP-090)" Sections 5.5 and 5.6.

Response:

Participants will be told they can discuss participation in research with their physician. Participants will also be allowed to go home to review the consent form and come back if they are still within 10 days of injury.

28.4 Describe any process to ensure ongoing consent, defined as a subject's willingness to continue participation for the duration of the research study.

Response:

If the participant return for their follow-up visit and has been performing the exercises at home, then ongoing consent will be implied.

28.5 Indicate whether you will be following "SOP: Informed Consent Process for Research (HRP-090)." Pay particular attention to Sections 5.4-5.9. If not, or if there are any exceptions or additional details to what is covered in the SOP, describe:

- *The role of the individuals listed in the application who are involved in the consent process*
- *The time that will be devoted to the consent discussion*
- *Steps that will be taken to minimize the possibility of coercion or undue influence*
- *Steps that will be taken to ensure the subjects' understanding*

Response:

☒ We have reviewed and will be following "SOP: Informed Consent Process for Research (HRP-090)."

Non-English Speaking Subjects

☒ **N/A:** This study will not enroll Non-English speaking subjects. *(Skip to Section 28.8)*

28.6 Indicate which language(s) other than English are likely to be spoken/understood by your prospective study population or their legally authorized representatives.

NOTE: The response to this Section should correspond with your response to Section 8.4 of this protocol.

Response:

28.7 If subjects who do not speak English will be enrolled, describe the process to ensure that the oral and written information provided to those subjects will be in that language, how you will ensure that subjects are provided with a sufficient period of time to consider taking part in the research study, and any process to ensure ongoing consent. Indicate the language that will be used by those obtaining consent.

NOTE: Guidance is provided on "SOP: Informed Consent Process for Research (HRP-090)."

Response:

Cognitively Impaired Adults

☒ **N/A**: This study will not enroll cognitively impaired adults. *(Skip to Section 28.9)*

28.8 Describe the process to determine whether an individual is capable of consent.

Response:

Adults Unable to Consent

☒ **N/A**: This study will not enroll adults unable to consent. (*Skip to Section 28.13)*

When a person is not capable of consent due to cognitive impairment, a legally authorized representative should be used to provide consent (Sections 28.9 and 28.10) ***and, where possible, assent of the individual should also be solicited*** *(Sections 28.11 and 28.12).*

28.9 Describe how you will identify a Legally Authorized Representative (LAR). Indicate that you have reviewed the "SOP: Legally Authorized Representatives, Children, and Guardians (HRP-013)" for research in New York State.

NOTE: Examples of acceptable response includes: verifying the electronic medical record to determine if an LAR is recorded.

Response:

☐ We have reviewed and will be following "SOP: Legally Authorized Representatives, Children, and Guardians (HRP-013)."

28.10 ***For research conducted outside of New York State****, provide information that describes which individuals are authorized under applicable law to consent on behalf of a prospective subject to their participation in the research. One method of obtaining this information is to have a legal counsel or authority review your protocol along with the definition of "legally authorized representative" in "SOP: Legally Authorized Representatives, Children, and Guardians (HRP-013)."*

Response:

28.11 Describe the process for ***assent of the adults****:*

- *Indicate whether assent will be obtained from all, some, or none of the subjects.* ***If some, indicate which adults will be required to assent and which will not.***

Response:

- ***If assent will not be obtained from some or all subjects, provide an explanation of why not.***

Response:

*28.12 Describe whether **assent of the adult** subjects will be documented and the process to document assent.*

NOTE: The IRB allows the person obtaining assent to document assent on the consent document using the "Template Consent Document (HRP-502)" Signature Block for Assent of Adults who are Legally Unable to Consent.

Response:

Subjects who are not yet Adults (Infants, Children, and Teenagers)

☒ **N/A**: This study will not enroll subjects who are not yet adults. *(Skip to Section 29.0)*

*28.13 Describe the criteria that will be used to determine **whether a prospective subject has not attained the legal age for consent to treatments or procedures involved in the research** under the applicable law of the jurisdiction in which the research will be conducted **(e.g., individuals under the age of 18 years)**. For research conducted in NYS, review "SOP: Legally Authorized Representatives, Children, and Guardians (HRP-013)" to be aware of which individuals in the state meet the definition of "children."*

NOTE: Examples of acceptable responses include: verification via electronic medical record, driver's license or state-issued ID, screening questionnaire.

Response:

*28.14**For research conducted outside of New York State**, provide information that describes which persons have not attained the legal age for consent to treatments or procedures involved the research, under the applicable law of the jurisdiction in which research will be conducted. One method of obtaining this information is to have a legal counsel or authority review your protocol along the definition of "children" in "SOP: Legally Authorized Representatives, Children, and Guardians (HRP-013)."*

Response:

28.15 Describe whether parental permission will be obtained from:

Response:

☐ One parent even if the other parent is alive, known, competent, reasonably available, and shares legal responsibility for the care and custody of the child.

☐ Both parents unless one parent is deceased, unknown, incompetent, or not reasonably available, or when only one parent has legal responsibility for the care and custody of the child.

☐ Parent permission will not be obtained. A waiver of parent permission is being requested.

NOTE: The requirement for parent permission is a protocol-specific determination made by the IRB based on the risk level of the research. For guidance, review the "CHECKLIST: Children (HRP-416)."

28.16Describe whether permission will be obtained from individuals ***other than parents****, and if so, who will be allowed to provide permission. Describe your procedure for determining an individual's authority to consent to the child's general medical care.*

Response:

28.17 Indicate whether assent will be obtained from all, some, or none of the ***children****. If assent will be obtained from some children, indicate which children will be required to assent.*

Response:

28.18 When assent of children is obtained, describe how it will be documented.

Response:

29.0 Waiver or Alteration of Consent Process

Consent will not be obtained, required information will not be disclosed, or the research involves deception.

☒ **N/A:** A waiver or alteration of consent is not being requested.

29.1 If the research involves a waiver or alteration of the consent process, please review the "CHECKLIST: Waiver or Alteration of Consent Process (HRP-410)" to ensure that you have provided sufficient information for the IRB to make the determination that a waiver or alteration can be granted.

NOTE: For records review studies, the first set of criteria on the "CHECKLIST: Waiver or Alteration of Consent Process (HRP-410)" applies.

Response:

29.2 If the research involves a waiver of the consent process for planned emergency research, please review the "CHECKLIST: Waiver of Consent for Emergency Research (HRP-419)" to ensure you have provided sufficient

information for the IRB to make these determinations. Provide any additional information necessary here:

Response:

30.0 Process to Document Consent

☐ **N/A:** A Waiver of Consent is being requested.
(Skip to Section 31.0)

30.1 Indicate whether you will be following "SOP: Written Documentation of Consent (HRP-091)." If not or if there are any exceptions, describe whether and how consent of the subject will be obtained including whether or not it will be documented in writing.

NOTE: If your research presents no more than minimal risk of harm to subjects and involves no procedures for which written documentation of consent is normally required outside of the research context, the IRB will generally waive the requirement to obtain written documentation of consent. This is sometimes referred to as 'verbal consent.' Review "CHECKLIST: Waiver of Written Documentation of Consent (HRP-411)" to ensure that you have provided sufficient information.

If you will document consent in writing, attach a consent document with your submission. You may use "TEMPLATE CONSENT DOCUMENT (HRP-502)". If you will obtain consent, but not document consent in writing, attach the script of the information to be provided orally or in writing (i.e. consent script or Information Sheet).

Response:

A consent form is attached.

☒ We will be following "SOP: Written Documentation of Consent" (HRP-091).

31.0 Multi-Site Research (Multisite/Multicenter Only)*

☒ **N/A:** This study is not an investigator-initiated multi-site study. This section does not apply.

31.1 Indicate the total number of subjects that will be enrolled or records that will be reviewed across all sites.

Response:

31.2 If this is a multi-site study ***where you are the lead investigator****, describe the processes to ensure communication among sites, such as the following.*

- *All sites have the most current version of the IRB documents, including the protocol, consent document, and HIPAA authorization.*

- *All required approvals have been obtained at each site (including approval by the site's IRB of record).*
- *All modifications have been communicated to sites, and approved (including approval by the site's IRB of record) before the modification is implemented.*
- *All engaged participating sites will safeguard data as required by local information security policies.*
- *All local site investigators conduct the study appropriately in accordance with applicable federal regulations and local laws.*
- *All non-compliance with the study protocol or applicable requirements will be reported in accordance with local policy.*

Response:

31.3 Describe the method for communicating to engaged participating sites.

- *Problems (inclusive of reportable events)*
- *Interim results*
- *Study closure*

Response:

31.4 If this is a multicenter study ***where you are a participating site/investigator****, describe the local procedures for maintenance of confidentiality.*

- *Where and how data or specimens will be stored locally?*
- *How long the data or specimens will be stored locally?*
- *Who will have access to the data or specimens locally?*
- *Who is responsible for receipt or transmission of the data or specimens locally?*
- *How data and specimens will be transported locally?*

Response:

31.5 If this is a multicenter study and subjects will be recruited by methods not under the control of the local site (e.g., call centers, national advertisements) describe those methods. Local recruitment methods are described elsewhere in the protocol.

- *Describe when, where, and how potential subjects will be recruited.*
- *Describe the methods that will be used to identify potential subjects.*
- *Describe materials that will be used to recruit subjects. (Attach copies of these documents with the application. For*

advertisements, attach the final copy of printed advertisements. When advertisements are taped for broadcast, attach the final audio/video tape. You may submit the wording of the advertisement prior to taping to preclude re-taping because of inappropriate wording, provided the IRB reviews the final audio/video tape.)

Response:

32.0 Banking Data or Specimens for Future Use*

☐ **N/A:** This study is not banking data or specimens for future use or research outside the scope of the present protocol. This section does not apply.

32.1 If data or specimens will be banked (stored) for ***future use, that is, use or research outside of the scope of the present protocol****, describe where the data/specimens will be stored, how long they will be stored, how the data/specimens will be accessed, and who will have access to the data/specimens.*

NOTE: Your response here must be consistent with your response at the "What happens if I say yes, I want to be in this research?" Section of the Template Consent Document (HRP-502).

NOTE: If the UBIRB has approved this study to bank data and/or specimens for potential future use outside the scope of this research study, any future use or disclosure of the data that is not described within the approved study must be submitted for review to the UBIRB.

Response: Data will be kept in the office of Dr. McPherson. The limited dataset will be kept in the same office or within a secure cloud storage drive (e.g., UB Box). These will be stored for 10 years or until all related projects have been completed. Dr. McPherson and the research team highlighted in this document will have access.

32.2 List the data to be stored or associated with each specimen.

Response:

Participant demographics, information from weekly participant check-ins, tests and measures completed by participants, data collected from EMR system including dates of service.

32.3 Describe the procedures to release banked data or specimens for future uses, including: the process to request a release, approvals required for release, who can obtain data or specimens, and the data to be provided with specimens.

Response:

The dates of service will be removed from any data shared with other researchers, thereby de-identifying the dataset. Thede-identified data will be made available to study personnel upon written or electronic request. No specimens to be collected. All future use of data from this study outside the scope of this protocol will be submitted to the IRB for review.
